# Correlated States in Strained Twisted Bilayer Graphenes Away from the Magic Angle

**DOI:** 10.1021/acs.nanolett.1c04400

**Published:** 2022-04-06

**Authors:** Le Zhang, Ying Wang, Rendong Hu, Puhua Wan, Oleksandr Zheliuk, Minpeng Liang, Xiaoli Peng, Yu-Jia Zeng, Jianting Ye

**Affiliations:** † College of Physics and Optoelectronic Engineering, Shenzhen University, Shenzhen 518060, China; ‡ Device Physics of Complex Materials, Zernike Institute for Advanced Materials, University of Groningen, 9747AG Groningen, The Netherlands; § CogniGron (Groningen Cognitive Systems and Materials Center), University of Groningen, 9747AG Groningen, The Netherlands

**Keywords:** Twisted bilayer graphene, electronic correlations, heterostrain, moiré superlattice, valley polarization

## Abstract

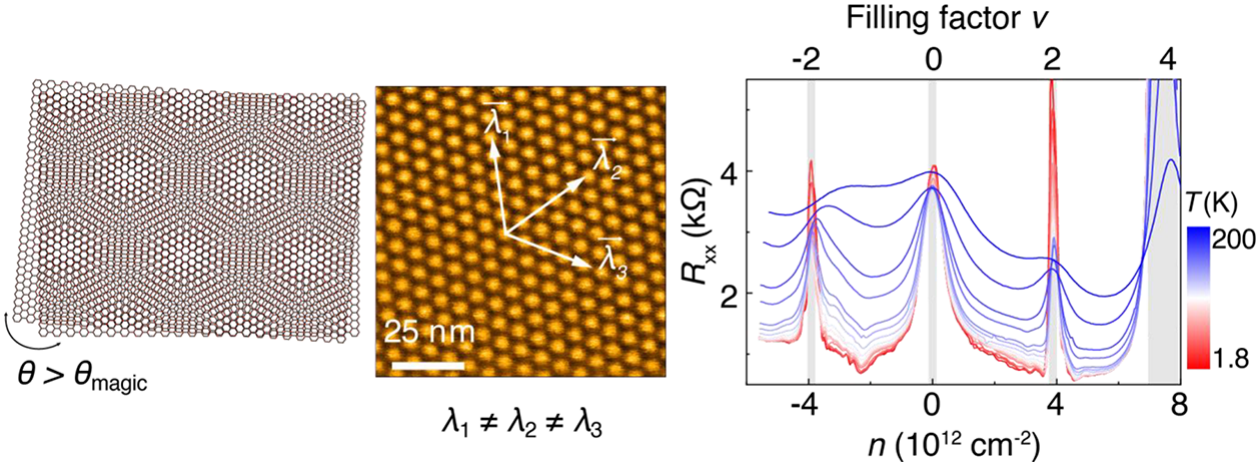

Graphene moiré superlattice formed by rotating two graphene sheets can host strongly correlated and topological states when flat bands form at so-called magic angles. Here, we report that, for a twisting angle far away from the magic angle, the heterostrain induced during stacking heterostructures can also create flat bands. Combining a direct visualization of strain effect in twisted bilayer graphene moiré superlattices and transport measurements, features of correlated states appear at “non-magic” angles in twisted bilayer graphene under the heterostrain. Observing correlated states in these “non-standard” conditions can enrich the understanding of the possible origins of the correlated states and widen the freedom in tuning the moiré heterostructures and the scope of exploring the correlated physics in moiré superlattices.

Strongly correlated systems can host diverse exotic quantum phases ranging from high-temperature superconductivity to fractional quantum Hall effect. Recently, the experimental realization of moiré flat bands in van der Waals heterostructures opens up new possibilities in studying the correlation effects.^1,2^ In particular, a famous example is the ultraflat bands formed in twisted bilayer graphene (TBG) due to interlayer coupling when two individual graphene sheets are rotated by the so-called “magic angle” of θ ≈ 1.1°.^3^ When these narrow bands are partially filled, denoted by the filling factor ν corresponding to the number of electron/hole per moiré unit cell, the Coulomb interaction dominates over kinetic energy. This gives rise to a wide variety of correlated states such as correlated insulator,^4−6^ symmetry-broken Chern insulator,^7^ and quantum anomalous Hall effect.^8,9^ Furthermore, the exotic electronic states can be easily accessed and tuned via electrostatic gating. This is due to the much lower carrier density required to fill a moiré unit cell compared with that required to fill the unit cell of a bare graphene. Meanwhile, the strength of electron–electron interaction in TBG can be further modified by adjusting the nearby screening environment, revealing a competition between correlated insulating states and superconductivity.^10−13^ In addition to the mechanism of doping a Mott insulator, recent observations of linear-in-temperature resistivity in TBG suggest a dominance of electron–phonon scattering,^14^ leaving the origin of the superconductivity in TBG an unsettled puzzle.

Besides rotating to the magic angle, the flatness of the moiré bands can be alternatively controlled by other parameters. For example, the electrical field can tune the flatness of isolated bands in twisted double-bilayer graphene (TDBG),^15−18^ and hydrostatic pressure can tune the interlayer-coupling strength in TBG,^6^ leading to new Coulomb-driven phases. Furthermore, buckling graphene onto an ultraflat substrate can also form flat bands without twisting.^19^ The buckling transition facilitates a periodic pseudomagnetic field, which can reconstruct the low-energy band into a set of mini flat bands.^20,21^

Beyond the methods above, an experimentally less explored way is to use the strain effect to prepare flat bands in moiré heterostructures. The heterostrain, that is, the relative strain between the top and bottom graphenes, widely exists in TBG and is generally considered detrimental to electrical transport.^22^ Nevertheless, recent spectroscopic experiments and theoretical works both showed that a slight uniaxial strain could induce flat bands in TBG.^23−26^ According to the continuum model, the Fermi velocity vanishes in the flat band.^27^ Thus, localized electrons amplify the effect of electron–electron or electron–phonon interaction, forming a series of correlated states (CSs). However, observing these CSs is somewhat limited at the magic angle. Recent experiments show that the CSs become much weaker at angles different from the magic angle.^28−30^ Therefore, it is highly demanded to have a practical tuning knob, such as the heterostrain, so that CSs can exist in broader twist angles.

We employed conducting atomic force microscopy (*c*-AFM) and transport measurements to investigate the influence of heterostrain on TBGs. Figure 1a shows the schematics of the *c*-AFM characterization on the TBG devices (see Supporting Information for methods). Exact moiré lattices are resolved by measuring the tunneling current between the *c*-AFM probe and the AA/AB sites of TBG flakes, allowing us to visualize the moiré superlattice up to hundreds of nanometers. Ideally, when the two graphene lattices rotate rigidly at a small angle without considering the strain or disorder, a single moiré period is expected for the superlattice. However, a small amount of strain is always present during the stacking and transferring processes, which causes the moiré lattice constant along the principal directions to be slightly different. Figure 1b, e, and h show current mappings of TBGs at three representative angles, that is, above, around, and below the magic angle. The bright spots are the high current region corresponding to the AA sites, while the dark regions are the AB/BA sites. Figure 1c shows typical current intensity profiles measured along marked directions shown in Figure 1b, yielding three different moiré lattice constants, λ = 6.8, 7.0, and 7.8 nm. Strained moiré lattices were also measured in Figures 1e and h, where λ = 12.9, 13.4, and 14.0 nm for Figure 1f, and 17.3, 17.8, and 18.4 nm for Figure 1i. The moiré lattice distortion is also clearly shown in the Fourier transform of real-space current mappings. As illustrated in Figures 1d, g, and j, the Bragg peaks after the Fourier transfer of the current mapping are different in three directions. In all measured TBGs, superlattice distortion induced by heterostrain exists ubiquitously. As a result, we determine the moiré lattice constant λ as an average of the different lattice constants found in three different directions marked by λ_1_–λ_3_. The twist angle θ follows λ = *a*/2 sin(θ/2), where *a* is the lattice constant of graphene. Besides the present metastable states, in small twist angles, θ ≈ 0, it is well-known that domain-wall-like boundary lines appear due to the superlattice reconstruction, which minimizes the heterostrain.^31,32^

**Figure 1:**
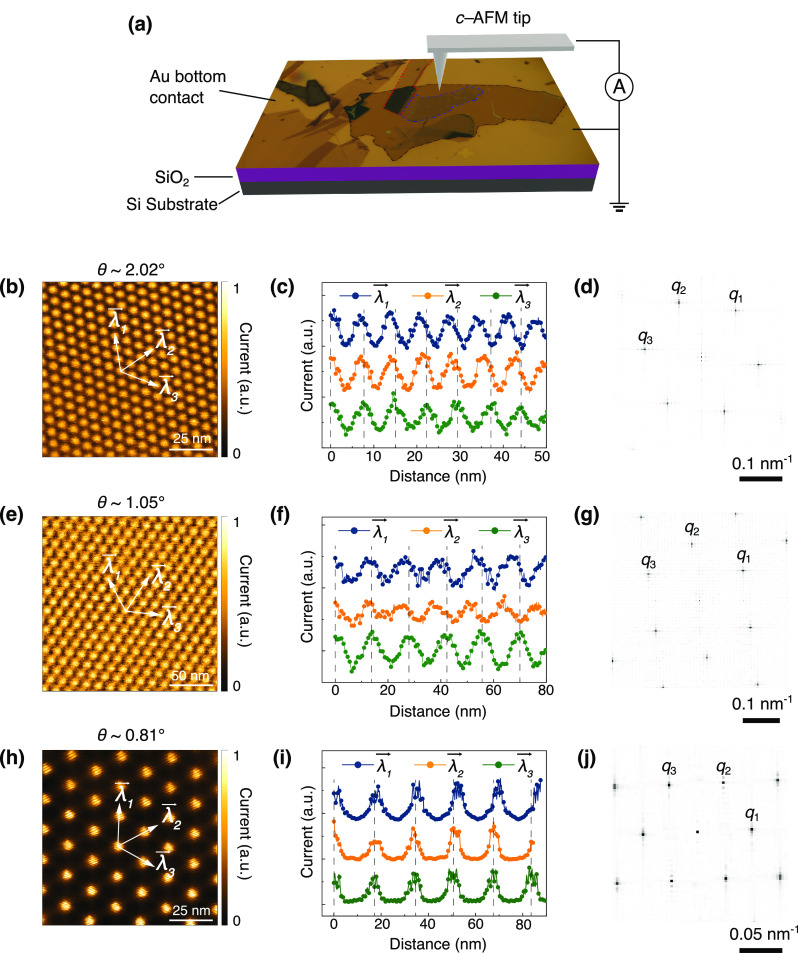
Visualization lattice distortion of moiré superlattices. (a) Schematic of the *c*-AFM measurement. An open TBG/ *h*-BN stack is laminated on an Au/SiO_2_/Si substrate. Black, blue, and red dash lines indicate the bottom *h*-BN, TBG, and graphite for grounding to the gold bottom contact, respectively. (b–j) Current mapping (panels in the left column), line profiles in different directions (middle column), and corresponding Fourier transfer (right column) of (b–d) larger than, (e–g) equal to, and (h–j) smaller than the magic angle TBGs. The moiré lattice constants along the principal directions are labeled as λ_1_ to λ_3_. The corresponding Bragg peaks are labeled from *q*_1_ to *q*_3_.

We then move to the transport measurement to observe the signature of heterostain. We fabricated encapsulated TBGs with a similar set of twist angles larger, close to, and smaller than the magic angle (θ = 1.83°, 1.65°, 1.1°, and 0.91°, respectively). Making sandwiched TBGs with bottom graphite gate utilized the “cut & stack” method (see Supporting Information for methods). Figure 2a shows the schematics of *h*-BN/TBG/*h*-BN/Graphite configuration with heterostrain, yielding three non-equal moiré wavelengths. The uniaxial heterostrain is directly visible in the moiré wavelengths along three directions in c-AFM characterizations (Figures 1b,e,h). On the other hand, the biaxial heterostrain is found in bubbles or wrinkles formed in making the stacks. We have selected a bubble-free region to define the channel, excluding the biaxial heterostrain as much as possible. Therefore, the heterostrain in the present study is most likely uniaxial. The existence of heterostrain in the encapsulated devices can be determined by the spatial inhomogeneity characterized by the carrier density require to form band insulating or correlated insulating states (Figure S1).^6^

**Figure 2:**
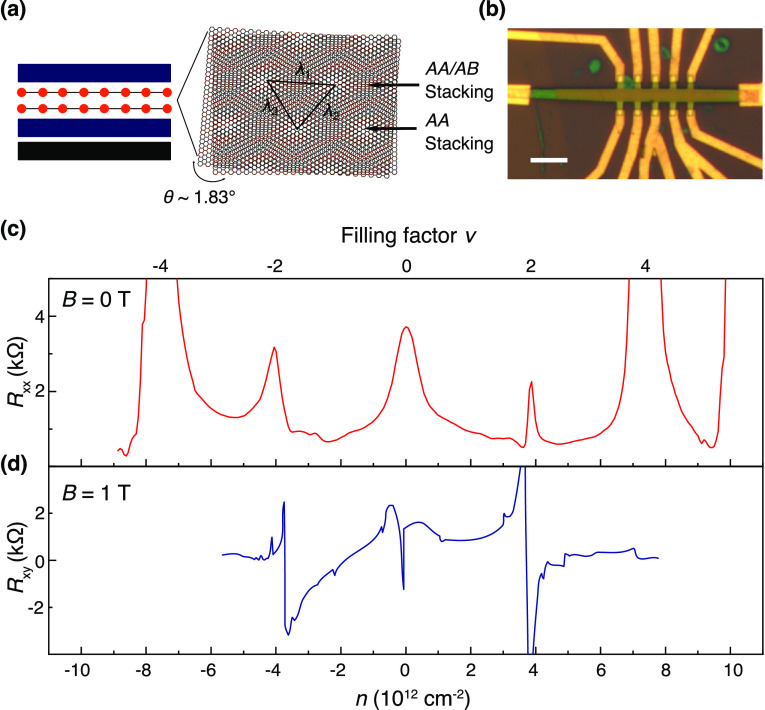
Structure of Device A and its transport characterizations. (a) Schematic of TBG sandwiched by *h*-BN (dark blue) layers. The TBG is gated by the bottom graphite (black). The TBG superlattice unit cell shows three nonequal moiré wavelengths λ_1_ to λ_3_. (b) Optical image of Device A, the scale bar is 5 μm. (c) Longitudinal resistance *R*_*xx*_ as a function of carrier density *n* measured at *B* = 0. The top axis is the carrier density normalized to the band filling factor ν. In this scan, we pushed the filling to cover ν = ± 4. Most of the reversible gating scans of Device A are performed within a safe range from −6 × 10^12^ cm^–2^ to 8 × 10^12^ cm^–2^, where the transfer characteristics are highly reversible. (d) Hall resistance *R*_*xy*_ measured at *B* = 1 T.

Figure 2b is an optical image of Device A with a bottom graphite gate to reduce gating inhomogeneity. The detailed transport measurement mainly focuses on Device A with θ = 1.83°, which is larger than the magic angle. Similar measurements in Device B (θ = 1.1°), C (θ = 0.91°), and D (θ = 1.65°) are shown in Figures S2 and S3. The gate-induced 2D carrier density is measured by the Hall effect around the charge neutrality point (CNP), which is also calibrated by the field effect by measuring the 2D Hall carrier density *n*_H_ = −*B*/(*eR*_*xy*_) at a low magnetic field. Figure 2c shows the four-terminal longitudinal resistance *R*_*xx*_ of Device A as a function of carrier density. Two symmetric insulating states appear at *n* = ± 7.5 × 10^12^ cm^–2^. The state at *n* = +7.5 × 10^12^ shows a clear semiconducting behavior, where the *R*_*xx*_ decreases with the increase of current excitation (Figure S4a). We can extract a gap of 46.2 meV by analyzing the thermally activated conductivity. These insulating states can be assigned as band insulators (BIs) of filling factor ν = ± 4 (see Figure S4b). The upturn of resistance with further electron filling corresponds to a higher energy-dispersive band, which is beyond our reach due to the limited achievable carrier doping. At lower filling, two well-developed insulating states emerge at ν = ± 2, where the TBGs with similar twist angles are either featureless^33^ or show weak correlation features.^28,29,34^ The corresponding Hall resistance measured at magnetic field *B* = 1 T (Figure 2d) shows apparent sign reversals at ν = 0, ± 2, and +4, when the hole-like pockets switch to the electron-like ones. The change in Fermi surface topology is due to a gap opening when the doping level crosses a Van Hove singularity, manifesting in the quantum oscillation (to be discussed later). In Device B, close to the magic angle, we also observed correlated insulating states at all integer fillings (Figure S2a,b). In Device C, smaller than the magic angle, we can again resolve a correlated insulating state at quarter-filling and half-filling (Figure S2c,d).

We further measured the longitudinal resistance *R*_*xx*_ versus carrier density at different temperatures for Device A (Figure 3). In magic-angle TBG (see high-temperature transport of magic-angle TBG shown in Figure S2b), all gaps emerge below 50 K, whereas all insulating peaks in Device A persist up to 200 K. The gap of the insulating states can be estimated in the Arrhenius plot of *R*_*xx*_ (Figure 3b), yielding gaps of 0.45 (ν = 2) and 0.22 meV (ν = −2). These gap sizes are close to those found in correlated insulators states of magic-angle TBG.^4−6^ The same analysis at the CNP finds a gap of 0.05 meV. The gap is small but remains consistent with the prediction that a gapped state exists at neutrality point for a wide range of twisting angles and interaction strengths.^5,35^ Between filling factor ν = 0 and −2, there exists an abrupt resistance drop, which is further analyzed. Figure 3c shows the temperature dependence of *R*_*xx*_ at several fixed carrier densities within ν = 0 and −2. At *n* = −2.3 × 10^12^ cm^–2^, the *R*_*xx*_ shows a *T*-linear behavior above 5 K and drops abruptly below 3 K. The linear relationship between *R*_*xx*_ and *T* is identical to what has been observed in magic-angle TBG^14,36^ and TDBG,^15,18^ indicating a phonon-mediated electron scattering process. The abrupt drop of resistance in TDBG,^16,18^ ABC-trilayer graphenes,^37^ and twisted bilayer WSe_2_^38^ was regarded as a signature of superconductivity. However, the recent results point to the origin of the Joule heating effect.^17^ When an out-plane magnetic field up to 6 T is applied, the drop of the *R*_*xx*_ cannot be entirely suppressed as shown in Figure 3d. Therefore, the possibility of having superconductivity as the cause can be ruled out. This drop in *R*_*xx*_ can be better described as a broad crossover, likely caused by charge-carrier scattering from ferromagnetic ordering,^39,40^ even though a clear signature of anomalous hall effect is also absent in our device. Another possible mechanism is the reduced inelastic scattering in the topological sub-bands induced by the strong interaction.^7,41,42^ The exact ground states in our TBGs as a function of carrier filling are still unclear. Further research is necessary to understand the exact scenario.^41,43^

**Figure 3:**
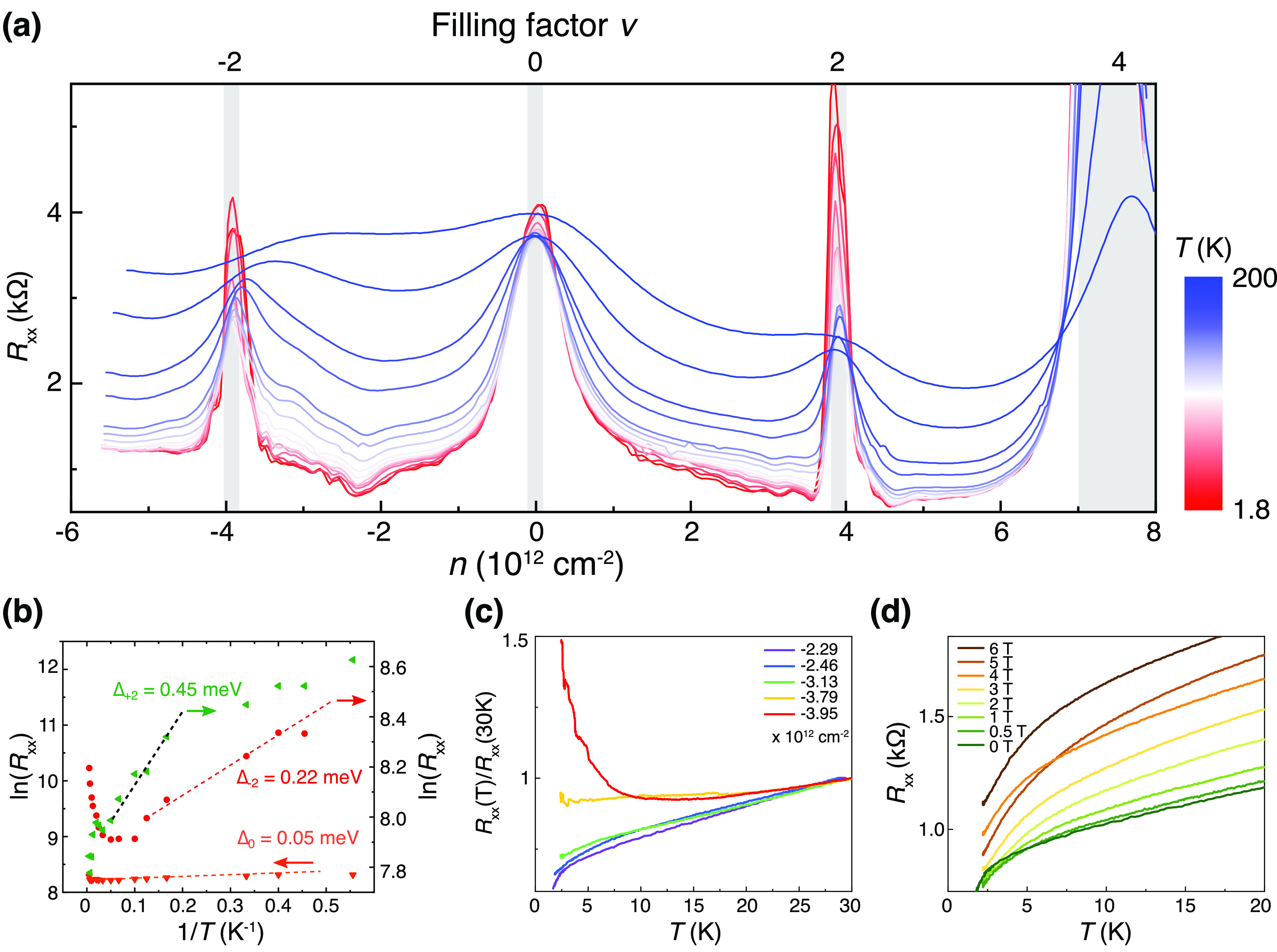
Correlated states in Device A, with an angle larger than the magic angle. (a) Longitudinal resistance *R*_*xx*_ as a function of carrier density *n* measured from 200 down to 1.8 K. (b) Arrhenius plot of *R*_*xx*_ at ν = 0, ± 2. The dash lines fit as *R*_*xx*_ ∝ exp(Δ/2*kT*) for thermal activation of conductivity, where Δ is the correlation-induced gap and *k* the Boltzmann constant. (c) Temperature dependences of *R*_*xx*_ for a few different carrier fillings between ν = 0 and ν = −2. The *R*_*xx*_–*T* dependences are normalized by the *R*_*xx*_ measured at 30 K. (d) *R*_*xx*_–*T* dependences for *n* = −2.3 × 10^12^ cm^–2^ are measured at different perpendicular magnetic fields.

When subjected to a perpendicular magnetic field, the Shubnikov-de Hass oscillation can reflect details of electron band structure such as the spin and valley degrees of freedom. Figure 4a shows the Landau fan diagram and corresponding trajectories originating from the different filling factors. Here, the Landau level filling factor ν_L_ is obtained from the Streda formula, ν_L_ = *nh*/(*eB*), where *h* is the Planck’s constant, and *e* is the elementary charge. In contrast to previous studies measured for the off-magic-angle devices, which show featureless Landau fans between CNP and BI, we observe a set of Landau fans originating from filling factor ν = 0, ± 2, 4. Around the CNP, we observe a 4-fold degenerate sequence of quantum oscillation, showing resistance minima at ν_L_ = ± 4, −8, 12, and ±16, as labeled in Figure 4b. An odd-number filling factor sequence due to the broken spin or valley symmetry is absent here, possibly due to the weaker electron–electron interaction that gets maximized at the magic angle.^32^ The quantum oscillation from ν = 2 exhibits 2-fold degeneracy. Combined with carrier density vanishing at ν = 2 extracted from Hall measurement as shown in Figure 2d, the ν = 2 band, composed of four spin/valley flavors, gets partially lifted due to interaction. The Landau levels emanating from ν = 0 and 4 filling positions appear at both higher and lower sides of the corresponding fillings, indicating that both electrons and holes contribute to the Landau fans. However, the Landau fans at ν = ± 2 positions extend away from CNP because the gaps at ν = ± 2 originate from the Coulomb exchange interaction between electrons, which emerges when the doping reaches half-filling. Therefore, the Landau fans extended only toward the higher carrier density, leaving the fans missing for the low carrier density side.^44,45^ Meanwhile, the *R*_*xx*_ values at ν = ± 2 monotonically increase with the increase of perpendicular magnetic field up to 14 T. This is in sharp contrast to the vanishing correlated insulating states measured in magic-angle TBG.^5,46^ Because of heterostrain, a pseudomagnetic field arises from the modulation of electron hopping due to lattice deformation. The pseudomagnetic field can polarize the spin to opposite directions in different valleys. An external out-plane magnetic field can induce valley-polarized states, which remain insulating at a high field, as shown in Figure 4c. These unique valley-polarized states in strained TBG are also observed in Device D (Figure S3). By combining the characterization of spatial inhomogeneity (Figure S1) and valley polarization, we confirm that strong CSs can exist in our off-magic angle systems under heterostrain.

**Figure 4:**
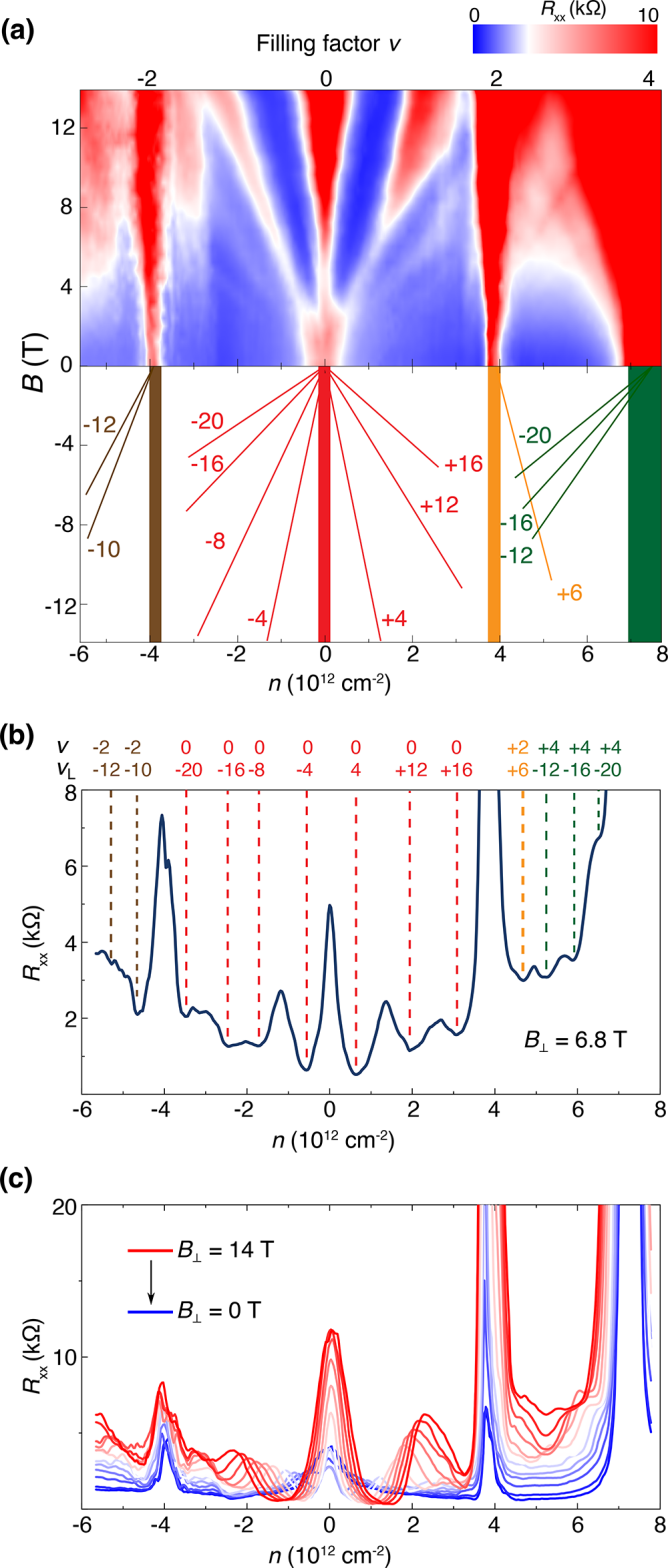
Quantum oscillation, Landau fans, and valley polarization states of Device A. (a) Upper, the contour plot of longitudinal resistance *R*_*xx*_ as a function of carrier density *n* and magnetic field *B*, measured at *T* = 1.8 K. Bottom, the schematics of the corresponding Landau level indices. The number labels the sequence of quantum oscillations emerging from ν = 0, ± 2, 4, respectively. (b) Line-cut of the panel along a fixed magnetic field *B* = 6.8 T. Dashed lines and indices of the band filling factor ν and Landau level filling factor ν_L_ are labeled for each resistance minimum. The dashed lines and ν, ν_L_ labels are color-coded, where colors are consistent with those used in labeling panel (a). (c) Line-cuts of panel (a) with magnetic field increasing from 0 to 14 T, in a step of 1 T.

Graphene is considered to have one of the most stable crystal structures due to the ultrastrong in-plane C–C bonding. Nevertheless, once the moiré superlattice is formed, the structure becomes metastable because of the superlubricity at the interface.^47^ As a result, rotation and even translation of graphene flakes after rapid thermal annealing have been observed in the graphene/*h*-BN superlattice. This apparent relaxation for the whole flake indicates that strain is distributed over a large area.^48^ In TBGs, the twisting angles between individual graphene layers can easily relax to zero or form large AB/BA domains, as shown in Figure S5. Furthermore, the relaxation remains active after the device fabrication. Evident changes in electronic properties can be observed after accumulating the relaxation process for a prolonged time. As shown in Figure 5a, in a freshly made Device E, the CSs emerge at ν = ±2, −3. After storage for 80 days under the ambient condition, all these CSs vanish, leaving blurred features (Figure 5b). The heterostrain increases the separation between the valence and conductions and sets a lower bound to the diminishing Dirac velocity, preventing it from vanishing at or around the magic angle.^26^ As a result, the strained TBG close to the magic angle becomes featureless in the carrier density dependence of *R*_*xx*_. By measuring the carrier density at the BIs, we can determine that the twisting angle decreases from 1.2° to 1.12° due to the presence of heterostrain, causing the relaxation of the TBG. This eventually results in more energy-favorable moiré superlattices of smaller angles.

**Figure 5:**
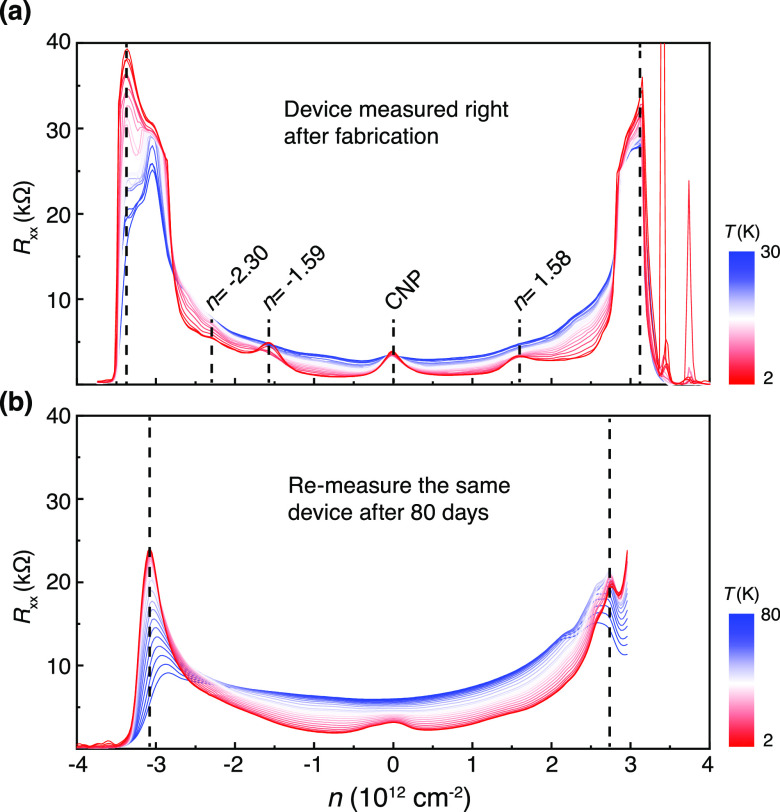
Relaxation of the moiré superlattice after storage at ambient condition. The longitudinal resistance *R*_*xx*_ as a function of carrier density *n* was measured at different temperatures (Device E, with an initial twisting angle of 1.2°, shows a twisting angle of 1.12° after relaxation). (a) Transport measurements performed right after the device fabrication. (b) Identical measurements performed after storing the device at ambient conditions for 80 days. We kept the scale identical in panels (a) and (b) to illustrate the relaxation effect. Black dash lines mark the positions of BIs. The gate offset values of CNP are offset by plotting the *R*_*xx*_ versus *n*.

In summary, using direct moiré visualization, transport characterization, and stability analysis confirms the existence of heterostrain in our TBG devices. The strained TBGs with various angles away from the magic angle can also host flat bands, manifesting as unexpected correlated states that are metastable under ambient conduction. Our results show an alternative path to create a moiré flat band in van der Waals heterostructures, which is yet to be fully explored. This finding would allow us to realize rich correlated electronic phases in moiré heterostructures.

## Supporting Information Available

Details of device fabrication and measurements, characterization of spatial inhomogeneity in Device A, correlated insulator states in Devices B and C, valley polarization states in Device D, feature assignment for band insulating state in Device A, visualization of atomic reconstruction under heterostrain in marginally TBG (PDF)
