# Supplementary material for: Correlated States in Strained Twisted Bilayer Graphenes Away from the Magic Angle
Source: Nano Lett. 2022 Apr 6;22(8):3204–11. doi: 10.1021/acs.nanolett.1c04400 (PMC9052762; doi:10.1021/acs.nanolett.1c04400)
Supplement: Supplementary file 1 — nl1c04400_si_001.pdf [file nl1c04400_si_001.pdf]

Supporting Information for

Correlated States in Strained Twisted-Bilayer Graphenes Away from the Magic Angle

Authors: Le Zhang[1,2], Ying Wang[2], Rendong Hu[2], Puhua Wan[2], Oleksandr Zheliuk[2,3], Minpeng Liang[2], Xiaoli Peng[2], Yu-Jia Zeng[1,*], Jianting Ye[2,*]

Affiliations:

[1] *College of Physics and Optoelectronic Engineering, Shenzhen University, Shenzhen, 518060, China*

[2] *Device Physics of Complex Materials, Zernike Institute for Advanced Materials, University of Groningen, 9747AG Groningen, The Netherlands*

[3] *CogniGron (Groningen Cognitive Systems and Materials Center), University of Groningen, 9747AG Groningen, The Netherlands*

[*]**Author to whom correspondence should be addressed**:

yjzeng@szu.edu.cn, j.ye@rug.nl

Contents:

Section 1: Device fabrication and measurements

Section 2: Characterization of spatial inhomogeneity in Device A

Section 3: Correlated insulator states in Device B and C

Section 4: Valley polarization states in Device D

Section 5: Feature assignment for the band insulating state in Device A

Section 6: Visualization of atomic reconstruction under hetero-strain in marginally TBG

Section 1: Device fabrication and measurements

Device fabrication and transport measurements. The monolayer graphene (lateral size larger than 50 μm), few-layers graphite (~4 nm thick), and h-BN flakes (thickness 30~40 nm) were all exfoliated on SiO_2 (285 nm)/Si substrate. We then performed a sequential "cut & stack" method to assemble the heterostructure.[1, 2] Before stacking, the graphene flake was cut using an atomic force microscopy tip. We used a tilted poly (bisphenol A carbonate) (PC)/PDMS stamp to pick up the top h-BN at 110 ℃, and carefully approached the h-BN to the cutting trace to pick up the first half of the graphene piece, rotated and picked up the second half. The three-layer stack was then laminated on a pre-prepared h-BN/bottom graphite substrate at 175 ℃. At this temperature. we gradually squeeze out the trapped interfacial bubbles before melting the PC.[3] The h-BN/bottom graphite substrate was made by the same dry transfer, followed by removing polymers with chloroform and isopropanol. The subsequent Argon annealing (atmospheric pressure, at 400 ℃ for 12 hours) removes the polymer residues. The stack was patterned by standard e-beam lithography and etched by CF_4 plasma. The graphenes are connected via edge contacts, composed of Ti/Au (2/80 nm) metal electrodes which is prepared by e-beam evaporation. We characterized the electrical transport of TBGs in a cryostat down to 1.8 K and up to 14 T. The R_{xx} and R_{xy} are measured by a pair of SR830 lock-in amplifiers using AC excitation smaller than 10 nA. The Keithley 2450 DC meters provide the DC voltage for gate bias.

***c*-AFM experiment**. We select large-area graphene for the stack. The open-top TBG sample is also fabricated using the "cut & stack" method. Instead of having full encapsulation, we sequentially pick up Graphene/Graphene/h-BN, then laminate the stack on the Au/SiO_2/Si substrate, leaving the top surface of TBG open for the AFM tips. The moiré pattern is then resolvable by the conducting AFM.

Twisting-angle determination. The filling factor v of the flat band can be calculated as $vn_0 = v A_0^{-1} = 4v(1 - cos\theta)/\sqrt{3}a^2$, where A_0 is the area of the unit cell of moiré superlattice, θ the twisting angle, and a the lattice constant of graphene.[4] For Device A, at v = ±2, the carrier density is $\pm 3.91\times10^{12}$ cm^{-2}. We can then determine the twisting angle θ = 1.83 ± 0.02°. Similarly, the

twisting angle of Device C and D are $\theta = 0.91 \pm 0.01°$ and 1.65°, respectively. For better comparison in Device E, the twisting angle is determined from carrier density at $v = 4$, which are -3.38×10^{12} and -3.06×10^{12} cm^{-2}, respectively. Therefore, we can determine that the twisting angle decreases from $\theta = 1.2°$ to 1.12°.

Section 2: Characterization of spatial inhomogeneity in Device A

The hetero-strain can change the effective area of the moiré unit cell for a given angle. Thus, unlike local compressibility and density of state measurements, the electrical transport measurement can only detect an averaged signal through many cells. However, measuring the carrier density of band or correlated insulators between different probes can determine inhomogeneity in the encapsulated sample. As shown in Figure S1, the two-probe conductance values are quite different by choosing different pairs of probes along the Hall bar. We have chosen the area that showed a clear difference in the required carrier density for different insulating states. The probe pairs C-D and D-E are selected as R_{xx} and R_{xy}, respectively. The spatial inhomogeneity is mainly caused by hetero-strain.

Section 3: Correlated insulator states in Device B and C

Considering both valley and spin degrees of freedom, the low-energy flat conduction and valence bands in first magic-angle TBG can be viewed as eight degenerate topological flat bands with opposite Chern number ±1.[5] When the bands are partially filled, the occupied Chern bands will be split from the unoccupied ones due to the long-range on-site Coulomb interaction, forming a gap at each integer filling. Our magic-angle TBG samples show well-separated correlated insulator states at each integer filling. Meanwhile, we also observed spatial inhomogeneity induced by hetero-strain. As shown in Figure S2b, the positions of band insulators gradually shrink, accompanying the vanishing correlated insulator states. In previous TBG samples with the twisting angle close to 0.9°, correlated insulator states are absent.[6] However, in our sample, we observed correlated insulator states at both quarter- and half-filling, which is consistently observed in the Hall effect measurement. Although the correlated insulator state at $v = -2$ is absent in Figure S2c, the sign reverse is evident in the Hall measurement as shown in Figure S2d, indicating that a

correlation-induced gap also forms at v= -2. Therefore, correlated insulator states exist in all of our strained TBG samples.

The flat band caused by the hetero-strain also induces very different correlated states, which were proposed and experimentally measured for different filling factors. Recently, two individual groups reported Pomeranchuk-effect in magic-angle TBG [7, 8]. This metallic behavior was also observed in the previous samples[9]. However, in our magic-angle TBG sample, insulating states at v= ±1 are robust. In the smaller angle sample, we can also observe the resistance increases at low temperature at v = 1, in contrast to the metallic behavior reported by other groups. Therefore, having a hetero-stain makes it difficult to conclude whether the ground state at a quarter filling is an actual iso-spin unpolarized phase or a disorder-related phase. The presence of hetero-strain is also highly likely to be the underlying reason for different behaviors found in similar measurements on samples of the same rotation angle prepared by different groups.

Section 4: Valley polarization states in Device D

Figure S3b shows two relatively weak resistance peaks at v = ±2, and the Hall measurement also shows two gaps at v = ±2. Figure S3d shows resistance at different magnetic fields, indicating valley polarization states here. The half-filling insulating states can persist up to 14 T. For a clear illustration, we only show the low-field data here because the quantum oscillations originating from CNP are more visible at high fields. The features observed in Device A and D are similar but different in magnitude. In the sandwiched sample, the structure of TBG is not accessible directly by the *c*-AFM or STM. Therefore, quantifying the strain directly in the sandwiched samples is yet to be achieved, which is crucial to determine the critical strain required to induce the correlated states in strained TBGs.

Section 5: Feature assignment for the band insulating state in Device A

We can observe semiconducting behavior when the Fermi level is in the bandgap between the flat conduction and higher energy-dispersive bands. As shown in Figure S4a, the resistance of band-insulating states increases with a weaker excitation. In addition, we can extract the activation gap

size from the temperature dependence of resistance up to 200 K. In the Arrhenius plot of R_{xx} (Figure S4b), we can extract a gap of 46.2 meV, similar to that found in larger angle TBGs.[10, 11] Thus, we can assign the feature at $n = 7.5 \times 10^{12}$ cm^{-2} to the band insulator, having a filling factor v =4.

Section 6: Visualization of atomic reconstruction under hetero-strain in marginally TBG

The TBG will undergo lattice reconstruction under the hetero-strain. The reconstruction effect is visible, especially when the twisting angle is close to 0°. Large domain-wall regions were also observed in our devices (Figure S5a). The overall morphology is similar to the previous visualization of the moiré superlattice by Piezo Force Microscopy (PFM)[12]. From the deformed lattice, we can estimate the moiré lattice constants to be ~47 and 188 nm, as shown in Figure S5 for panel a and b, respectively. This range of lattice constants corresponds to twist angles of θ = 0.3° (a) and 0.075° (b).

References

(1) Chen, G., et al. Evidence of a gate-tunable Mott insulator in a trilayer graphene moiré superlattice. *Nat. Phys.* **2019,** 15, (3), 237-241.

(2) Saito, Y., et al. Independent superconductors and correlated insulators in twisted bilayer graphene. *Nature Physics* **2020,** 16, (9), 926-930.

(3) Purdie, D. G., et al. Cleaning interfaces in layered materials heterostructures. *Nat. Commun* **2018,** 9, (1), 5387.

(4) Lu, X., et al. Superconductors, orbital magnets and correlated states in magic-angle bilayer graphene. *Nature* **2019,** 574, (7780), 653-657.

(5) Liu, J.; Liu, J.; Dai, X. Pseudo Landau level representation of twisted bilayer graphene: Band topology and implications on the correlated insulating phase. *Phys. Rev. B* **2019,** 99, (15), 155415.

(6) Arora, H. S., et al. Superconductivity in metallic twisted bilayer graphene stabilized by WSe2. *Nature* **2020,** 583, (7816), 379-384.

(7) Rozen, A., et al. Entropic evidence for a Pomeranchuk effect in magic-angle graphene. *Nature* **2021,** 592, (7853), 214-219.

(8) Saito, Y., et al. Isospin Pomeranchuk effect in twisted bilayer graphene. *Nature* **2021,** 592, (7853), 220-224.

(9) Yankowitz, M., et al. Tuning superconductivity in twisted bilayer graphene. *Science* **2019,** 363, (6431), 1059-1064.

(10) Polshyn, H., et al. Large linear-in-temperature resistivity in twisted bilayer graphene. *Nat. Phys.* **2019,** 15, (10), 1011-1016.

(11) Cao, Y., et al. Superlattice-Induced Insulating States and Valley-Protected Orbits in Twisted Bilayer Graphene. *Phys. Rev. Lett.* **2016,** 117, (11), 116804.

(12) McGilly, L. J., et al. Visualization of moire superlattices. *Nat. Nano.* **2020,** 15, (7), 580-584.

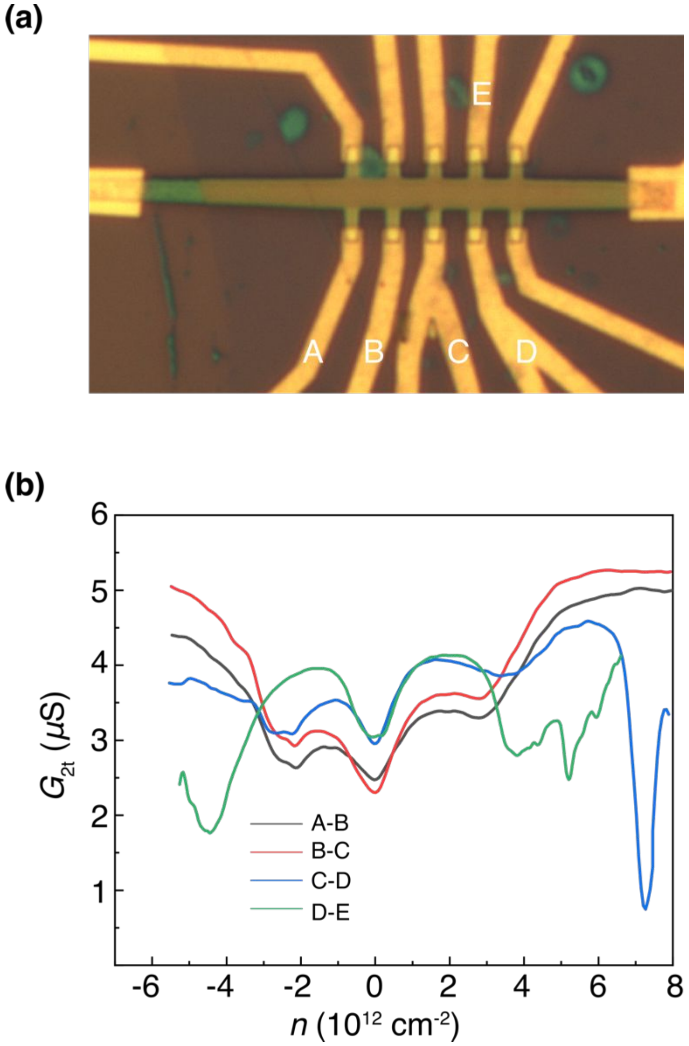

Figure S1. (a) Optical image of Device A with marked leads. (b) two-terminal conductance between different probes in Device A.

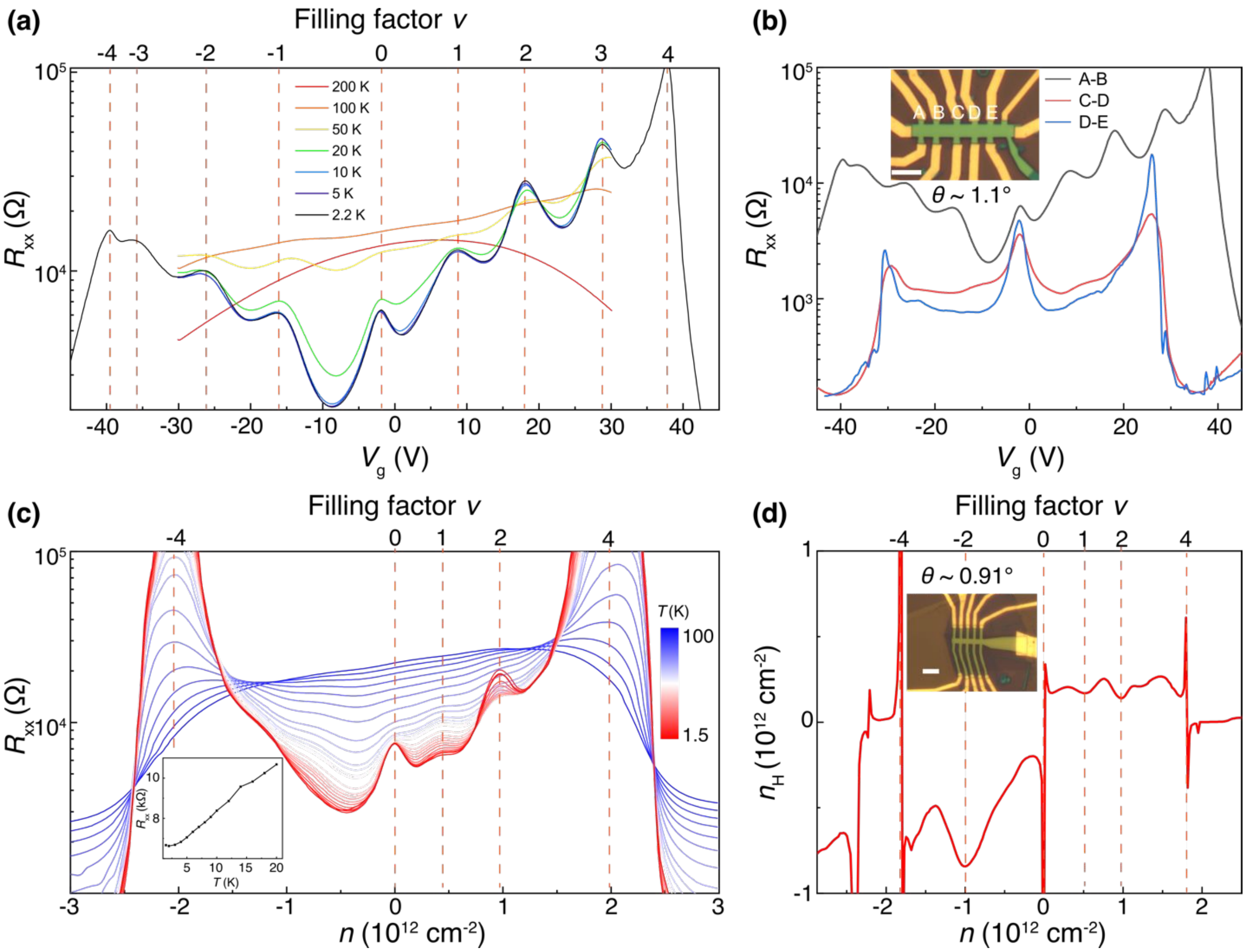

Figure S2. **Correlated insulator states in Device B and C** (a) Four-terminal longitudinal resistance as a function of gate voltage at variable temperatures from 1.5 to 200 K for Device B. (b) Four-terminal resistance measured between the marked probes, inset: the optical image of Device B with SiO_2 back gate, the scale bar is 5 μm. (c) Temperature-dependent resistance versus carrier density for Device C, inset: the temperature dependence of resistance at filling v = 1. (d) Hall carrier density $n_H = -B/eR_{xy}$ extracted from the low-field Hall resistance *versus* total gate-induced carrier density, inset: the optical image of Device C with graphite back gate, the scale bar is 5 μm.

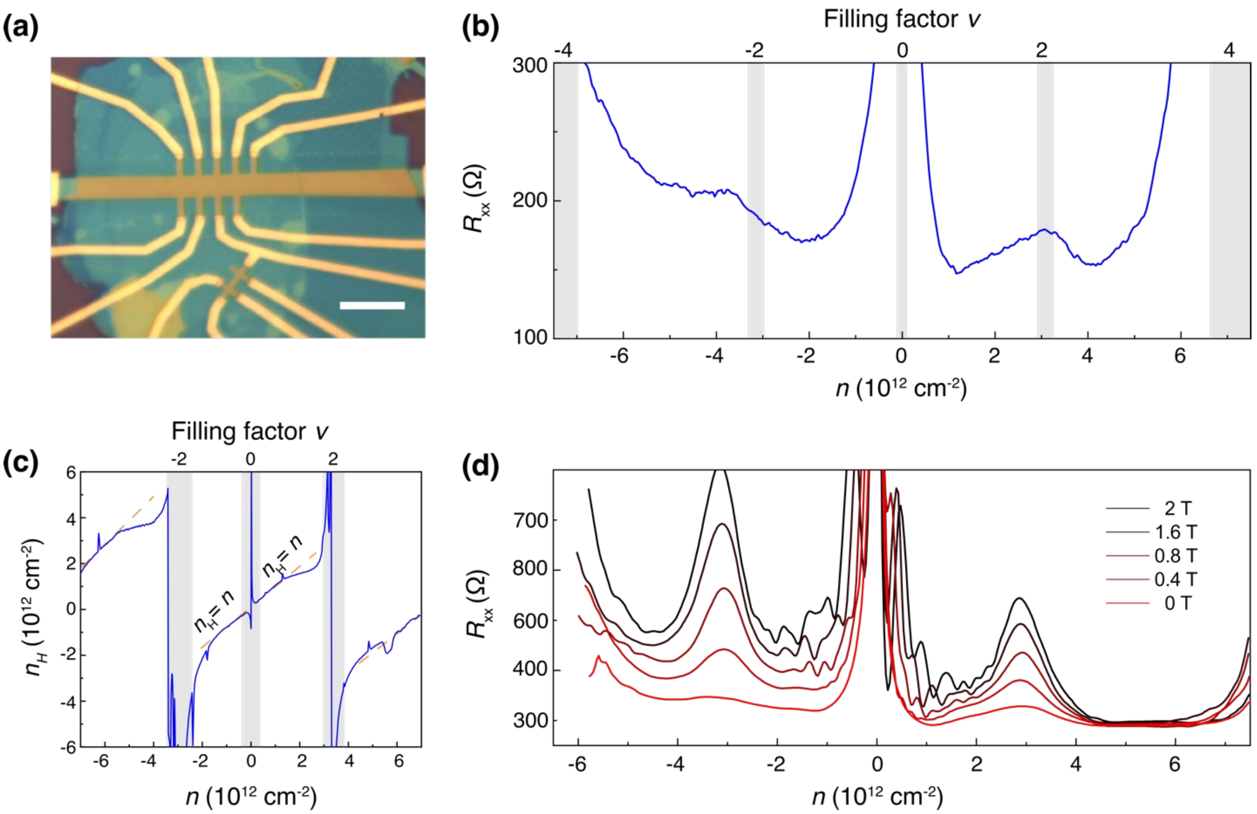

Figure S3. Valley polarization states in Device D (θ= 1.65°). (a) Optical image of Device D, the scale bar is 5 μm. (b) Longitudinal resistance versus carrier density at 1.8 K. The top axis is the carrier density normalized to the band filling factor ν. (c) Hall measurement at 1T. (d) Longitudinal resistance as a function of carrier density at a different perpendicular magnetic field.

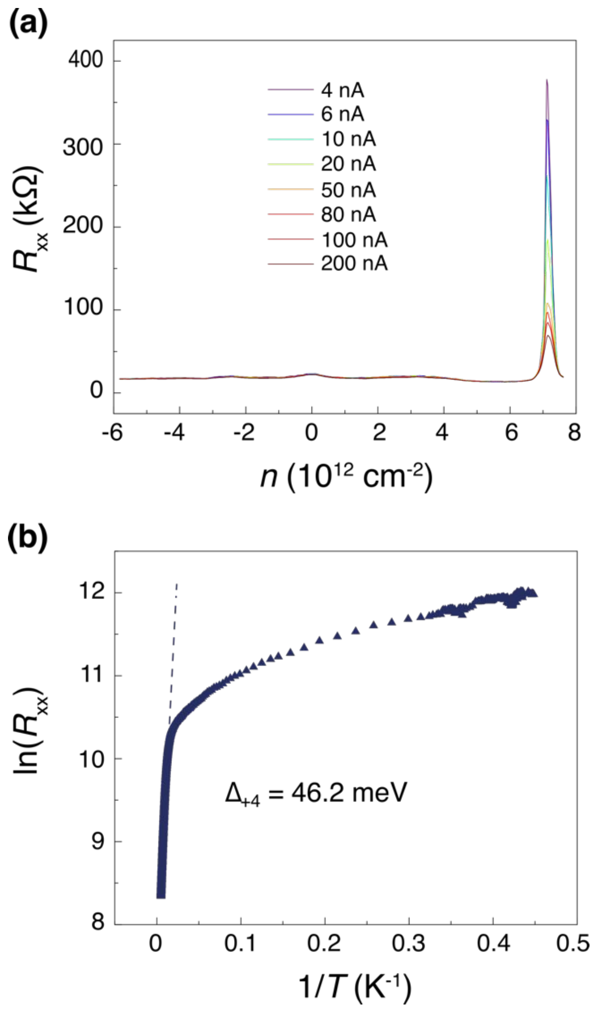

Figure S4. **Transport Characterization of the band insulator of Device A.** (a) Longitudinal resistance R_{xx} versus carrier density with different current excitation. (b) Arrhenius plot of resistance at n= 7.5 $\times 10^{12}$ cm^{-2} shows that the bandgap at the complete filling of ν = +4 is 46.2 meV.

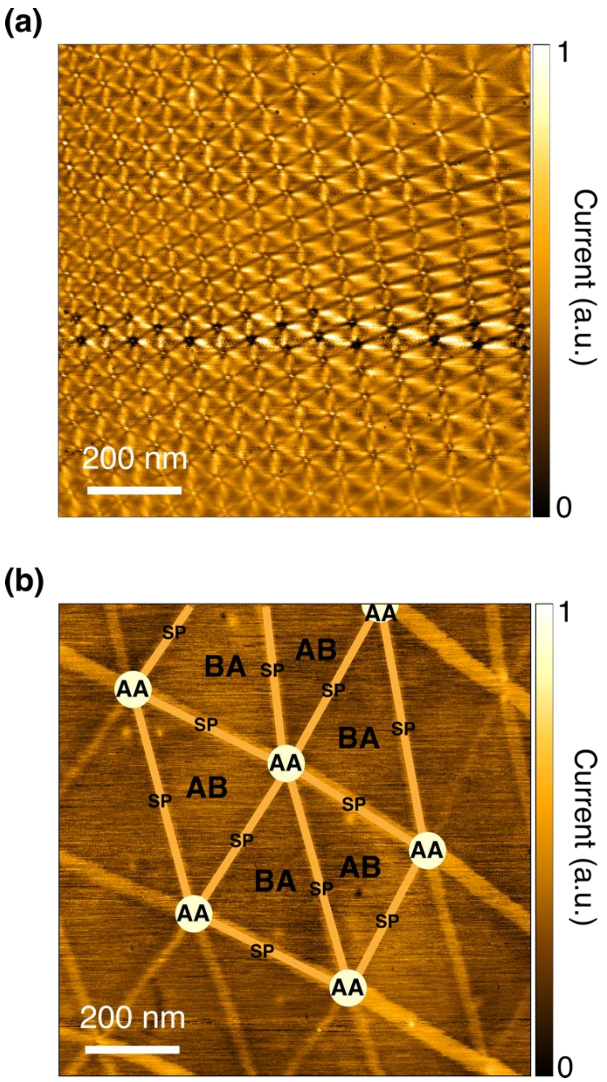

Figure S5. **TBGs with small twisting angles ~0°** (a) Lattice reconstruction under hetero-strain for a sample with a small twisting angle. (b) Expanded current mapping of a small angle ($\theta = 0$) TBG, showing the reconstructed AA sites, areas of AB/BA stackings, and saddle-point (SP) stackings.
